# Characterisation of a Spontaneous Mutant of *Lemna gibba* G3 (Lemnaceae)

**DOI:** 10.3390/plants12132525

**Published:** 2023-07-02

**Authors:** Lakshmi Pasricha Sarin, K. Sowjanya Sree, Károly Bóka, Áron Keresztes, Jörg Fuchs, Akhilesh K. Tyagi, Jitendra Paul Khurana, Klaus-Juergen Appenroth

**Affiliations:** 1 Department of Plant Molecular Biology, University of Delhi South Campus, New Delhi 110021, India; lakshmi.sarin@rajguru.du.ac.in (L.P.S.); akhilesh@genomeindia.org (A.K.T.); khuranaj@genomeindia.org (J.P.K.); 2 Department of Environmental Science, Central University of Kerala, Periye 671320, India or ksowsree9@cukerala.ac.in; 3 Department of Plant Anatomy, Eötvös Loránd University, H-1117 Budapest, Hungary; karolyboka@caesar.elte.hu (K.B.); keresztes.aron@ttk.elte.hu (Á.K.); 4 The Leibniz Institute of Plant Genetics and Crop Plant Research (IPK), 06466 Seeland, Germany; fuchs@ipk-gatersleben.de; 5 Matthias Schleiden Institute—Plant Physiology, University of Jena, 07743 Jena, Germany

**Keywords:** *Lemna gibba*, polyploidisation, spontaneous mutation

## Abstract

A spontaneous mutant of the duckweed *Lemna gibba* clone no. 7796 (known as strain G3, WT) was discovered. In this mutant clone, *L. gibba* clone no. 9602 (mt), the morphological parameters (frond length, frond width, root length, root diameter) indicated an enlarged size. A change in the frond shape was indicated by the decreased frond length/width ratio, which could have taxonomic consequences. Several different cell types in both the frond and the root were also enlarged. Flow cytometric measurements disclosed the genome size of the WT as 557 Mbp/1C and that of the mt strain as 1153 Mbp/1C. This represents the results of polyploidisation of a diploid clone to a tetraploid one. The mutant clone flowered under the influence of long day-treatment in half-strength Hutner’s medium in striking contrast to the diploid WT. Low concentration of salicylic acid (<1 µM) induced flowering in the tetraploid mutant but not in the diploid plants. The transcript levels of nuclear-encoded genes of the photosynthetic apparatus (*CAB*, *RBCS*) showed higher abundance in light and less dramatic decline in darkness in the mt than in WT, while this was not the case with plastid-encoded genes (*RBCL*, *PSAA*, *PSBA*, *PSBC*).

## 1. Introduction

Lemnaceae (waterlentils or duckweed) represent a small family of monocotyledonous, aquatic plants [1]. It consists of 36 species categorized into five genera, i.e., *Spirodela*, *Landoltia*, *Lemna*, *Wolffiella* and *Wolffia* [2]. Especially in the last decade [3] this plant family had unfolded a broad spectrum of potential practical applications. These range from phytoremediation of wastewater [4,5,6], to use for human nutrition and animal feeding [7,8,9,10], to the production of starch for bioalcohol conversion [11,12] and to biogas production [12]. Moreover, as a result of whole genome sequencing of increasing numbers of duckweed species and clones, e.g., in *Spirodela polyrhiza* [13,14], and the feasibility of application of other molecular methods such as genetic transformation using CRISPR/Cas9 [15], members of the Lemnaceae became “a model plant system in the genomics and postgenomics era” [16].

Although the members of Lemnaceae do undergo generative propagation through flowers and seeds, the most common mode of their growth and propagation is by vegetative means. Budding of daughter fronds from the vegetative pouch of the mother frond, producing clones of the mother is a common property of duckweeds [16]. On the way to becoming an unconventional crop plant, the natural variance of physiological and biochemical properties of these plants has to be investigated in order to prepare for biotechnological applications. Out of the several thousands of duckweed clonal accessions in the stock collections worldwide (International Steering Committee on Duckweed Research and Applications [17]), only a few dozens of them have been characterized concerning their growth rates or protein content. These investigations already suggest a huge natural variation between the clones or ecotypes of the same species. Apart from collecting a higher number of ecotypes of species, one other way to extend the available bioresource is to prospect for polyploid mutations in the already collected clones, either by scoring for spontaneous mutants or by artificially inducing mutations in the plants. The artificial induction of mutation was performed in *Landoltia punctata* (*Spirodela oligorrhiza*) which resulted in larger phenotypes with higher dry weight but without any changes in growth rates of the mutant plants when compared with the wild-type plants [18].

*Lemna gibba* G3 is a long-day strain, which has been extensively employed as a model system to artificially induce flowering [19,20,21]. In order to study the physiology of flowering, this strain was obtained from Charles F. Cleland (then at the Smithsonian Institution, Washington, DC, USA) in 1985–1986 by the late Jitendra P. Khurana (JPK) at the University of Delhi, South Campus and was maintained ever since under aseptic conditions. While rescuing the fronds of *L. gibba* G3 in late 1988 from an over-grown (3-month-old) agar culture, one of the colonies displayed an altered phenotype upon multiplication after being transferred to fresh liquid medium, i.e., it was considerably larger in size when compared to other colonies from the same culture. This putative mutant strain, which originated spontaneously, was transferred to fresh medium periodically and the progeny was found to retain the phenotype over several generations and has been maintained under axenic (“sterile”) conditions ever since. Subsequently, this mutant was found to flower under certain conditions in which its parent strain *L. gibba* G3 did not flower normally.

In a previous study too, a mutant was resurrected from primary callus cultures of strain G3 of *L. gibba* [22], with an enlarged frond size. The self-pollination of cultures indicated that this mutant strain, jsR_1_, contains essentially the same amount of DNA per nucleus as its parent line. However, a substantial increase in indole-3-acetic acid levels as estimated by GC-MS was recorded in the mutant in comparison to wild-type, and this tendency was observable at several stages of the culture raised under axenic conditions [22].

In the present work, we investigated the spontaneous mutant of *L. gibba* G3 (called throughout the manuscript as a mutant, mt) and compared it with the parental wild type (WT). As it turned out, the mt was generated by spontaneous polyploidisation, resulting in a tetraploid form. Morphological, anatomical, physiological and biochemical consequences of this genetic alteration were investigated, which included also the induction of flowering by salicylic acid and different daily light periods.

## 2. Results

**Figure 1:**
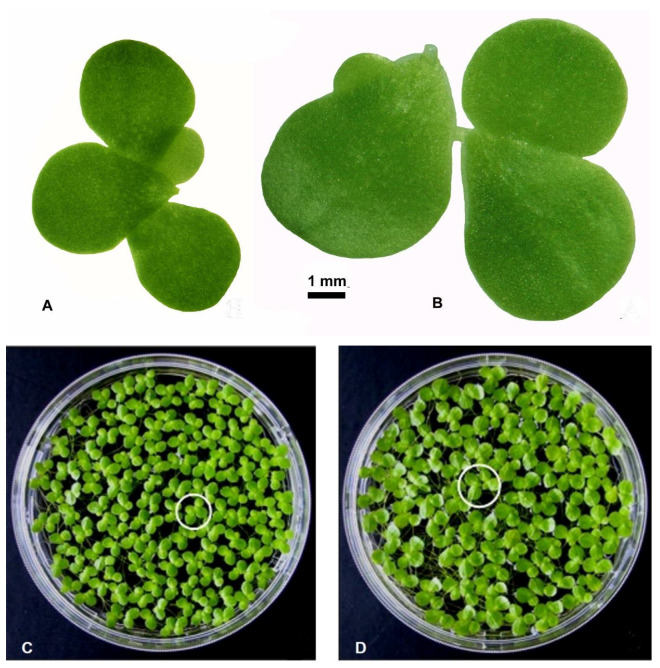
Fronds of the duckweed *Lemna gibba*. (**A**) Colony of the wild-type WT (clone number 7796) with mother frond, two daughter fronds and one granddaughter frond. (**B**) Colony of the mutant mt (clone number 9602), also with one mother frond, two daughter fronds, and one granddaughter frond. (**C**) Population of WT. (**D**) Population of mt, both in 9 cm Petri dishes. White circles focus on characteristic colonies.

The fronds of *L. gibba*, the parental wild type (WT, clone no. 7796) and the spontaneous mutant (mt, clone no. 9602) differed clearly in their morphology, strikingly visible concerning their frond size and frond shape. These differences are quite obvious at both the single colony and the population levels (Figure 1A–D). In Figure 1A,B, a representative colony of both WT and mt clones are shown. In each case, the colony depicts a mother frond, two daughter fronds and a granddaughter frond, connected by stipes. The biometric measurements showed that both the frond length and the frond width of mt clone were significantly larger than those of WT plants. These changes not only increased the size of the fronds in mt clone but also modified the length/width ratio of the frond. This ratio decreased in the mt clone as compared to the WT (Table 1). As a result, the shape of the frond was altered to being more obovate in mt clone than in WT clone. Differences were obvious concerning the root morphology as well. The mt possessed longer and thicker roots in comparison to those of WT (Table 1).

**Figure 2:**
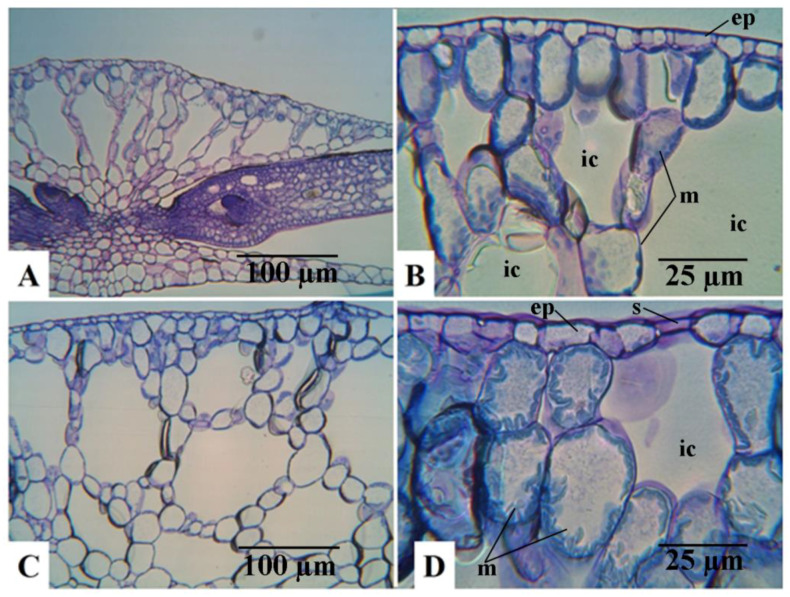
Micrographs of frond cross sections of *Lemna gibba* (**A**,**B**): WT 7796 and (**C**,**D**): mutant mt 9602 (ep: epidermis; ic: intercellular space; m: mesophyll cells; s: stoma).

**Figure 3:**
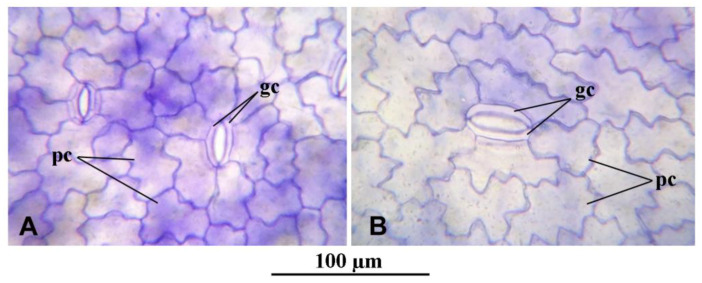
Micrographs of epidermal peels of dorsal epidermis of *Lemna gibba* showing stomatal guard cells (gc) and pavement cells (pc). (**A**): WT 7796, (**B**): mt 9602.

**Figure 4:**
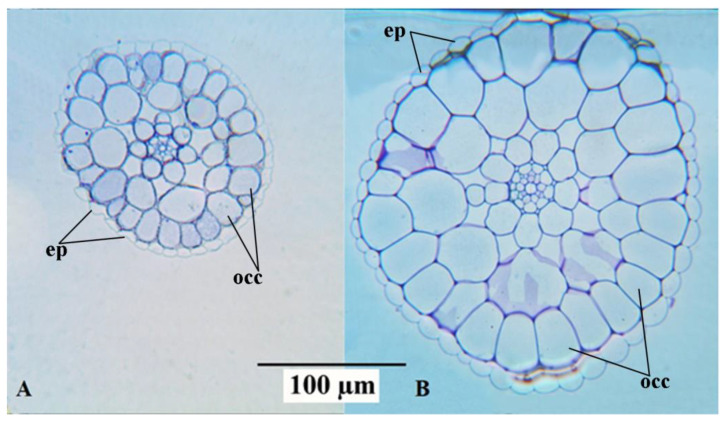
Micrographs of cross sections of roots of *Lemna gibba* (**A**): WT 7796, (**B**): mt 9602 (ep: epidermis; occ: outer cortical cells).

Anatomical observations were in accordance with the morphological biometric data. Light microscopic observations of the cross sections of fronds showed that the sub-epidermal cells or the mesophyll cells of the fronds were distinctly larger in mt than in the WT clone (Figure 2). The difference in cell size concerning the epidermal cells between the diploid and the tetraploid clones was less evident. This is mainly because of the large variability of these cells, as shown in the dorsal epidermis of the frond cross sections in both WT and mt (Figure 2B,D, respectively). The same is also shown in the light microscopic observations of the dorsal epidermal peels. The pavement cells of the dorsal epidermis were noted to be highly variable in shape and size in both clones (Figure 3A,B). However, the stomatal guard cells were more uniform and were significantly larger in the mt clone than in WT (Figure 3). Interesting observations were also made from the cross sections of roots of the WT and mt clones. It was noticed that the outer cortical cells in the root of mt were double the size of those in the root of WT (Table 1). Although the diameter of the root in clone mt was almost double that of clone WT (Table 1), the cross sections of the root showed that the number of root epidermal cells was equivalent at a similar position of the root (apical part) in both mt and WT clones. This indicates increased cell size of the root epidermal (atrichoblast) cells in mt in comparison to WT (Figure 4). The increase in cell size was also observed for all the endodermal cells in mt. In the root cross-sections, phloem and xylem cells also appeared to be larger in clone mt than in the WT clone (Figure 4).

**Figure 5:**
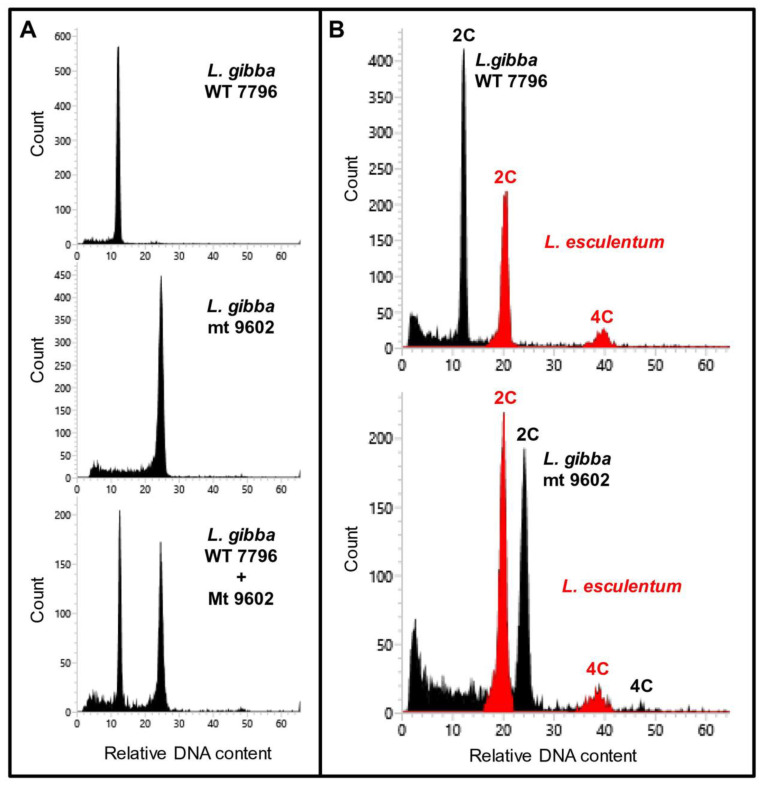
Flow cytometry confirmed the tetraploid status of the mutant clone 9602 in comparison to the diploid WT clone 7796. (**A**): Measurements of both clones separately or in combination clearly demonstrated different ploidy levels. (**B**): Genome size measurements using internal reference standards (exemplified here with *Lycopersicon esculentum* in red).

Genome size measurements were carried out for *L. gibba* WT and mt by flow cytometry using internal reference standards (Figure 5). The genome size of WT was found to be 557 Mbp per 1C and that of mt was 1153 Mbp/1C. This indicated doubling of genome size in the mt when compared to WT. For comparison, three other clones of *L. gibba* collected from different geographic regions of the globe were also analysed. All these three clones of *L. gibba* had similar genome sizes as the WT clone (Table 2). The detailed measurement data can be found in Supplementary Table S1.

The physiological capacity of the mutant clone (mt) in comparison to the parent clone (WT) was investigated in terms of both vegetative propagation and generative propagation. The vegetative propagation was measured in terms of relative growth rates (RGR) and the generative propagation was scored by the flower induction capacity of the clones. The RGR was determined under standard growth conditions (following otherwise the ISO 20079 [23]) in two different nutrient media, i.e., N- and half-strength Hutner´s medium (see Supplementary Material, Table S2). Both the clones performed better in N-medium when compared to half-strength Hutner´s medium. However, it was found that the mt clone grows significantly slower than the parental strain in both nutrient media used in this study (Table 3).

**Figure 6:**
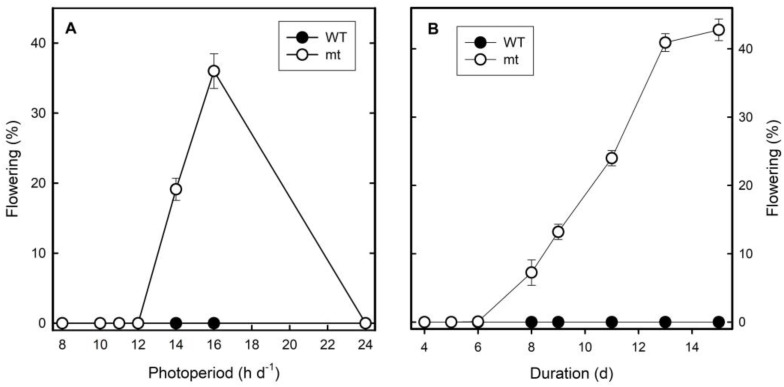
Influence of light periods on the flowering response of *Lemna gibba* WT 7796 and mt 9602. (**A**) Influence of photoperiod on flower induction. Cultures were pre-cultivated under non-inductive short days (8 h light:16 h dark) and thereafter subjected to appropriate photoperiods for 10 days, followed by 3 days of continuous light, and finally analysed for flowering. (**B**) Kinetics of induction of flowering in WT and mt under long days of 16 h light:8 h darkness. Single 4-frond colony was inoculated per flask and flowering was analysed on specified days. In all experiments, half-strength Hutner´s nutrient medium was used.

In the following experiments, the influence of photoperiod on flower induction was investigated in detail. Flowering was not detected under continuous light in both clones. Clone mt flowered up to 20 and 40% under 14 and 18 h light periods per day, respectively, but not at shorter photoperiods. Clone WT, however, did not flower under any of the light conditions investigated (Figure 6A). It was demonstrated that eight days of long-day treatments were required to induce flowering in clone mt and the intensity of response increased further by increasing the number of days of long-day treatments to 14 or 15 days. The WT, however, did not respond at all with respect to flowering to any of these treatments (Figure 6B).

**Figure 7:**
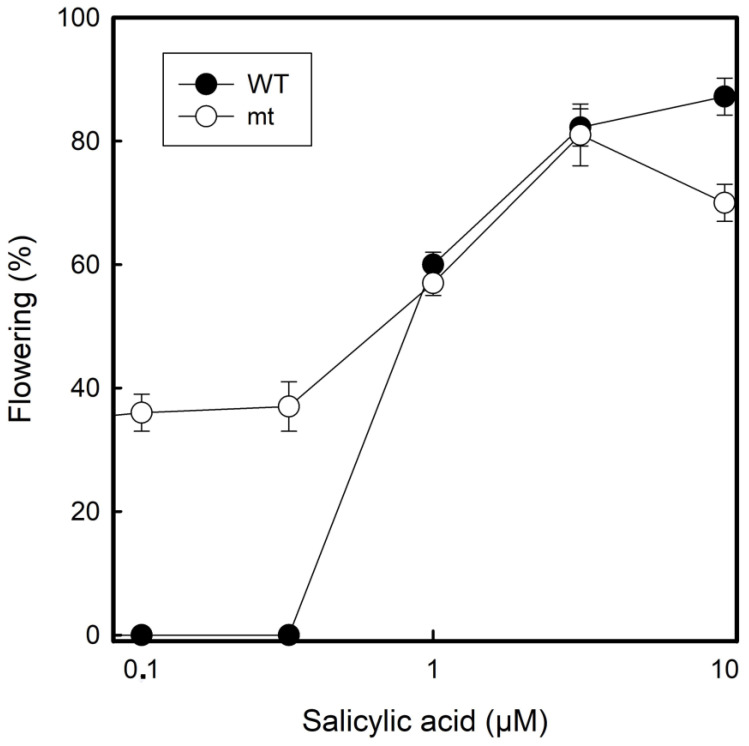
Effect of salicylic acid on flowering in *Lemna gibba* WT 7796 and mt 9602, grown in half-strength Hutner’s medium. The experimental cultures were initiated in salicylic acid-containing medium and analysed for flowering after 11 long days (16 h light:8 h dark).

Flower induction was investigated under the influence of salicylic acid as has been investigated before with the same species and the same clone 7796 under long-day conditions (16 h light: 8 h darkness) [24]. At concentrations ≥1 µM of salicylic acid, both WT and mt, responded in a similar manner. However, there was a striking difference at lower salicylic acid concentrations (0.1 µM and 0.5 µM). The WT did not flower at all under these conditions, but the mt flowered to approximately 30%. At higher salicylic acid concentrations (10 µM), both the clones performed with ca. 80% flowering frequency (Figure 7).

Biochemical properties were investigated and compared between both clones. Most of the parameters, chlorophyll a, carotenoid and protein content were lower in clone mt on a dry weight basis. However, dry weight and chlorophyll b content did not show any significant differences between the two clones (Table 4).

**Figure 8:**
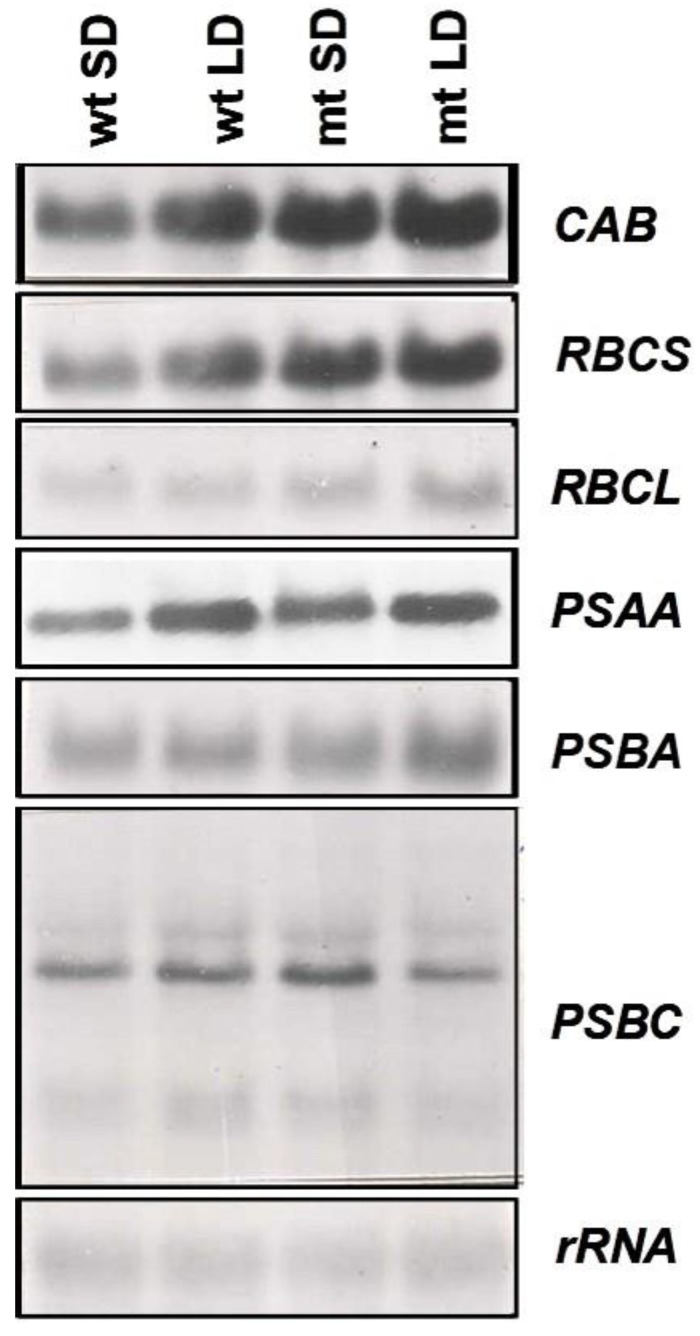
Changes in the steady-state transcript levels of nuclear (*CAB*, *RBCS*) and plastid (*RBCL*, *PSAA*, *PSBA*, *PSBC*) encoded genes of the photosynthetic apparatus in the wild-type and the mutant strain of *L. gibba* G3 grown in ½ Hutner’s medium under long day and short day conditions. Note that the mutant strain was flowering under the LD conditions.

To examine the changes in expression of the genes of the photosynthetic apparatus, the changes in transcript levels of two nuclear-encoded (*CAB*, *RBCS*) and four plastid-encoded (*RBCL*, *PSBA*, *PSAA* and *PSBC*) genes were examined. An increase in steady-state transcript levels of the nuclear-encoded genes, *CAB* and *RBCS*, was observed in the mutant as compared to the wild-type and the expression was more under long-day conditions than short-day conditions (Figure 8) suggesting the influence of duration of light on the expression of these genes. Much less pronounced changes were observed for the plastid-encoded genes (*RBCL*, *PSAA*, *PSBA* and *PSBC*). The rDNA probe was used as a loading control and was found to be satisfactory as is evident from the autoradiogram presented in Figure 8.

**Figure 9:**
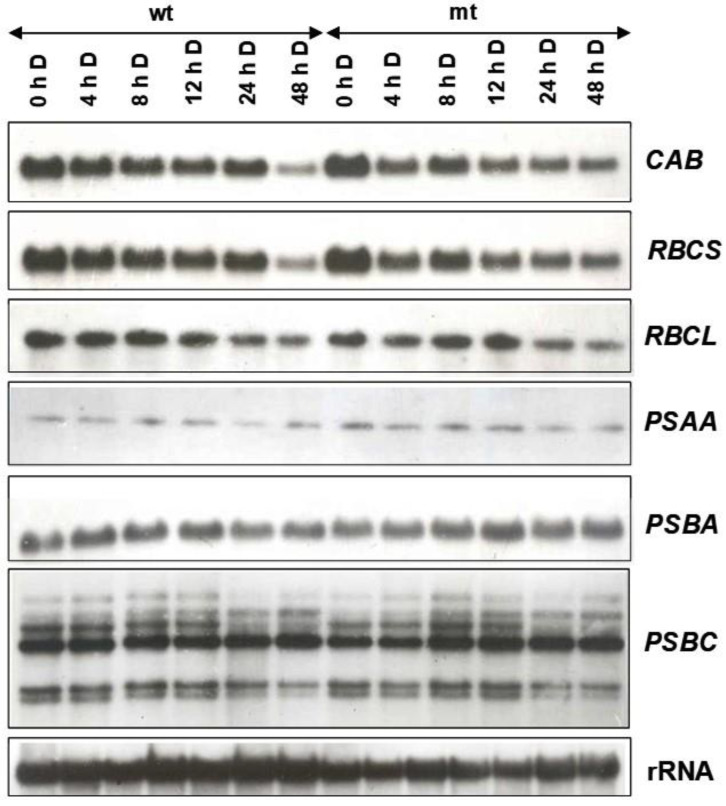
Dark adaptive changes in expression of nuclear (*CAB*, *RBCS*) and plastid (*RBCL*, *PSAA*, *PSBA*, *PSBC*) encoded genes of the photosynthetic apparatus in the wild-type and the mutant strain of *L. gibba* G3. Both the strains were initially grown under non-inductive short days for 15 cycles and then transferred to continuous light for 2 d, before subjecting them to dark adaptation for specified time (between 4 to 48 h). Total RNA was extracted, and northern blots were prepared. Northern analysis was performed using heterologous probes. For checking the quality and quantity of RNA, rRNA probe was used.

To find out if the lower content of chlorophyll in the mutant may be accounted for due to changes in expression pattern in the mt due to dark adaptation, the fronds of the wild-type and the mutant were initially grown under short day (so that both of them remain vegetative) for 15 cycles and then transferred to continuous light for two days before being subjected to dark adaptation. As is obvious from the autoradiogram in Figure 9, the transcript levels of the nuclear-encoded genes (*CAB* and *RBCS*) were somewhat higher in the mutant grown in continuous light and those of plastid-encoded genes showed no significant difference in the transcript abundance. On dark adaptation, the transcript levels of nuclear-encoded genes declined but remained somewhat steady up to 24 h and, in the following 24 h in darkness, a sharp decline was noted in the wild-type, whereas mutant showed relatively less drastic decline under similar conditions (Figure 9). In contrast to nuclear genes, plastid-encoded genes did not show much fluctuation in their transcript abundance, both in the mutant and the wild-type. However, most of the transcripts encoded by the *PSBC* operon did show a decline in dark adaptation (Figure 9). Strikingly, the transcript of *PSBC* operon displayed an increase in dark adaptation for 8 h or more, in both WT and mt.

## 3. Discussion

The genome size of the two investigated clones of *L. gibba* showed that the WT 7796, also known as *L. gibba* G3, has a similar genome size as several other clones of this species (Table 2). Urbanska-Worytkiewicz [25] and Geber [26] investigated chromosome numbers of many duckweed clones including those belonging to *L. gibba* and reported values of 2n = 40, 42, 44, 50, 70, 80 and 84 for this species. These data were cited by Wang et al. [27] without critical evaluation. However, the technique was not yet much developed at that time and the results should be treated with care, considering the high chromosome number variation within the same species [25]. Comparing all available genome sizes and chromosome number measurements [25,26,28], the most probable chromosome number in *L. gibba* is 2n = 40 [28], defining the clone WT 7796 as a diploid. Consequently, the spontaneously formed mutant, mt 9602 is a tetraploid and might be an autotetraploid as the mutation proceeded in a closed Erlenmeyer flask of axenic (“sterile”) cultures of the clone WT 7796. Theoretically, there are two mechanisms of how genome duplication could have happened. First of all, during vegetative propagation, the mitotic division between two S phases could have failed. Alternatively, two gametes of the same clone could be non-reduced. However, this second mechanism of self-fertilisation by spontaneously non-reduced gametes is very improbable considering also the low flowering frequency in this clone/species. To the best of our knowledge, this is the first report about spontaneous polyploidisation in the family of Lemnaceae. Vunsh et al. [18] reported polyploidisation in *L. punctata*, induced by treatment with colchicine that interferes with cell divisions [29]. Intraspecific fertilisation between different clones of a species has been reported in *Lemna aequinoctialis* (termed *Lemna paucicostata*; [30]) and in *L. gibba* [24]. However, no polyploidisation was observed in these cases.

Artificial polyploidisation is often used to improve plant quality, e.g., of ornamental plants in horticulture [29] and is generally used in plant breeding [31]. Moreover, polyploidisation (e.g., genome duplication) steps were important events during the evolution of plants. Often polyploidisation process results in plants with specific properties, e.g., larger leaves or fruits. In agreement with this general expectation, in the present work, the tetraploid plants were characterized by an increase in the size of several of the investigated morphological and anatomical parameters, such as frond and root size as well as the size of the cells in frond and root.

Morphologically, duckweeds are small in size, ranging from a centimetre to less than a millimetre. As a consequence, the morphological markers available for delineation of the different duckweed species, especially within a genus are scarce and often confusing for generalists. One of the features observed between the WT and mt clones investigated in this study was that the disproportionate increase in frond length and frond width had altered the shape of the frond in mt, thereby making it more obovate than the fronds of WT. Such changes in frond shape are noteworthy, especially in view of the fact that the above-mentioned biometric parameters play a significant role as morphological markers for the determination of duckweed species [2]. Hence it is suggested to use the morphological markers with care and caution and to use molecular markers for barcoding [16] wherever necessary for Lemnaceae species determination.

As a general physiological parameter, RGR was investigated. Unexpectedly, the RGR of the tetraploid clone 9602 was significantly lower than the diploid parent clone, both in N- and in half-strength Hutner´s medium. Notably, the decreased growth capacity correlated with the biochemical results: the chlorophyll a (but not chlorophyll b) and the carotenoid content were significantly lower in the tetraploid mt (clone 9602) than in the diploid WT parent clone (7796). Evidently, the photosynthetic capacity became partly impaired during polyploidisation. In accordance with these results, also the protein content, as a biochemical cross-parameter for the metabolic capacity, was lowered in the tetraploid clone.

The photoperiodic response of flower induction in *L. gibba* was already investigated by Kandeler [32]. He mainly used clone G1 but mentioned that clone G3 (the diploid clone investigated in the present paper) behaved very similarly. According to his results, *L. gibba* is a long-day plant with a critical day-length of 12 to 14 h. Cleland and Briggs [33,34] reported an increase in light sensitivity of flower induction in *L. gibba* G3 about 8 to 10 h after the start of the light period. In the present paper, both WT and mt *L. gibba* clones were investigated under different light regimes for 10 days. The mt did respond to these regimes in terms of flowering, unlike the WT. The critical day length was between 12 and 16 h. No flowering was observed in continuous light. Extending the duration of irradiation enhanced the effect on flowering until ca. 13 days and any further extension showed a saturated effect.

Exposure to salicylic acid is more effective in inducing flowering than different light periods alone. Here, the tetraploid mutant mt 9602 is more sensitive. While the wild type did not flower at low salicylic acid concentrations (<1 µM), the tetraploid mutant did flower to approximately 40%. Fu et al. [24] investigated the same WT clone of *L. gibba* (also clone G3, but registered in the duckweed stock collection of Elias Landolt in Zurich additionally as clone 7741) and found that application of salicylic acid is absolutely required to induce flowers and to produce seeds. However, these authors applied only concentrations of ≥10 µM salicylic acid. The whole genome of this clone has now been sequenced [35].

This difference in the response of WT and mt to flower induction is of interest for future studies on the probable sexually propagated generations of this mt clone of *L. gibba*. The genome sequencing of these clones can unravel more details on polyploidisation and can open further avenues.

The investigation of the transcript levels in WT and mt showed higher expression of the investigated nuclear-encoded genes (*CAB* and *RBCS*) in mt whereas the plastid-encoded genes (*RBCL*, *PSAA*, *PSBA* and *PSBC*) showed only minimal differences between both strains. During dark-adaption, the transcript levels of nuclear-encoded genes decreased even more dramatically in the mt whereas the plastidic-encoded transcripts showed no significant differences between the two strains. This does not explain immediately the lower chlorophyll content in mt. However, an imbalance between nuclear-encoded and plastid-encoded transcripts becomes evident and is even stronger in mt than in WT. It is difficult to explain the lower chlorophyll contents in mt than in WT considering the relative increase in abundance of the transcript, particularly of the nuclear-encoded genes, *RBCS* and *CAB,* and an altered rate of decline of transcripts during dark adaptation of mt, in comparison to its WT parent. It could be that the change in ploidy level in the mutant has caused a higher expression of nuclear-encoded genes in the mutant. However, this higher expression need not necessarily correlate with the level of the corresponding polypeptides that finally assemble in a definite ratio for establishing the photosynthetically competent chloroplast machinery.

## 4. Materials and Methods

### 4.1. Plant Material and Cultivation

*Lemna gibba* L., clone G3 was provided by Charles F. Cleland (then at the Smithsonian Institution, Washington, DC) in 1985–1986 to one of the authors (JPK) at the University of Delhi, South Campus, New Delhi, India. This clone is registered in many stock collections under several international IDs, e.g., 7796 at the University of Jena, Germany. In 1988, from a three-month-old culture, one of the authors (JPK) isolated a spontaneously formed mutant with a changed phenotype showing unusually large fronds and is now registered under the ID 9602. Throughout the manuscript, this mutant was indicated by “mt” whereas the parental, wild type is indicated by “WT”. Three more clones of *L. gibba* were obtained from the stock collection of the University of Jena and used for genome size measurements as controls.

During the investigations, plants were either cultivated in N medium [36] or in half-strength Hutner´s medium [37]. For the composition of the media see Supplementary Material, Table S2. The temperature during all experiments was maintained at 25 °C. The plants were exposed to light conditions provided by fluorescence tubes at an intensity of 100 µmol m^−2^ s^−1^.

### 4.2. Morphological-Anatomical Investigations

Clones 7796 (WT) and 9602 (mt) of *L. gibba* were investigated using a stereo microscope for morphological biometric studies. Anatomical investigations were carried out by light microscopic studies. Halved fronds and 1 cm long root pieces (apical part) were fixed in 4% formaldehyde solution (in 0.05 M phosphate buffer, pH 7.2). After thoroughly washing with the same buffer, samples were dehydrated in an increasing ethanol gradient and embedded in Technovit 7100 resin (Kulzer GmbH, Wehrheim, Germany). From 5-5 blocks, 1 µm thick sections were prepared with glass knives using a Microm HM 360 microtome (Microm International Ltd., Walldorf, Germany). Sections were stained with toluidine blue and observed in Nikon Eclipse 80i microscope (Nikon Corp., Tokyo, Japan).

The dorsal epidermal peels were stained with toluidine blue and observed using 40× magnifying objective under a light microscope.

### 4.3. Flow Cytometric Genome Size Estimation

Genome size measurements were performed using either a BD Influx cell sorter (BD Biosciences, San Jose, CA, USA) or a CyFlow Space flow cytometer (Partec GmbH, Münster, Germany) according to Dolezel et al. [38]. Fronds were chopped with a razor blade together with young leaf material of *Raphanus sativus* ‘Voran’ (IPK gene bank accession number RA 34; 2C = 1.11 pg; [39]), *Lycopersicon esculentum* Mill. convar. *infiniens* Lehm. var. *flammatum* Lehm. ‘Stupicke Rane‘ (IPK gene bank accession number: LYC 418) or *Glycine max* cv. Cina 5202 ‘Voran’ (IPK gene bank accession number SOJA 392; 2C = 2.23 pg; [40]) as internal reference standard.

For isolation of nuclei and their staining, either Galbraith’s buffer [41] supplemented with DNAse-free RNase (50 µg/mL) and propidium iodide (50 µg/mL) or the DNA staining kit ‘CyStain^R^ PI Absolute P’ (Partec GmbH, Münster, Germany) was used. Usually, 10,000 nuclei per sample were analysed and at least four independent measurements per clone were performed. The absolute DNA contents (pg/2C) were calculated based on the mean values of the G1 peak and the corresponding genome sizes (Mbp/1C) according to Dolezel et al. [42].

### 4.4. Relative Growth Rate Measurements

Measuring growth rates on the basis of frond number and calculating the relative growth rates (RGR, d^−1^), doubling time (DT, d) and relative weekly yield (RY, number of fronds after one week of cultivation starting with one gram fresh weight [43]) were performed as described earlier [44].

### 4.5. Induction of Flowering

Flowering was induced by different concentrations of salicylic acid applied directly to Hutner´s medium. During this treatment, long day conditions were applied (16 h light: 8 h dark) for 11 days. Thereafter, plants were de-stained in 70% ethanol overnight in glass tubes and flowering was evaluated under a stereo microscope. To investigate the influence of light periods, experimental cultures were initiated in half-strength Hutner’s medium with the transfer of one 4-frond colony from cultures maintained in the same medium, under non-inductive short days (8 h light: 16 h dark). Cultures were then subjected to appropriate photoperiods for 10 days, followed by 3 days of continuous light, and analysed for flowering as described above. Finally, plants were treated under long-day conditions (16 h light: 8 h dark) and the duration of treatments was increased from 4 to 15 days.

### 4.6. Analytical Methods

Freshly harvested plant material was dried for 24 h at 105 °C and weighed before drying and thereafter. Chlorophyll and carotenoid content were investigated by homogenisation of 150 mg frozen fresh material in a mortar with ammonia-buffered acetone (800 mL acetone + 195 mL water + 5 mL 25% ammonia), and centrifugation at 15,000× *g* in a fixed-angle rotor of a bench-top centrifuge for 15 min. The absorbance of the supernatant was measured at 663 nm, 647 nm and 470 nm in a UV/Vis spectrophotometer and the pigment content was calculated according to Lichtenthaler [45]. The protein content was measured by homogenisation of 150 mg frozen fresh material in a mortar with 10 mM KOH and centrifuged at 15,000× *g* for 15 min. A fraction of the supernatant (100 µL) was mixed with 5 mL Bradford reagent and measured in a UV/Vis spectrophotometer at 595 nm and 465 nm. The ratio A_595_/A_465_ was plotted against the concentration of BSA, and the protein content was calculated with the help of a linear regression line [46].

### 4.7. Isolation of Total RNA

Total RNA was isolated by the protocol described by Logemann et al. [47]. About two g of tissue was frozen in liquid nitrogen and homogenized to a fine powder with the help of a mortar and pestle. It was then transferred to SS 34 rotor tube, and suspended in two volumes of guanidine buffer (8 M guanidine hydrochloride, 20 mM MES, 20 mM EDTA, 50 mM 2-mercaptoethanol, pH 7.0) immediately after the evaporation of liquid nitrogen. The extract was then centrifuged at 10,000 rpm for 15 min. The supernatant was filtered through one layer of mira cloth and extracted with one volume of phenol: chloroform: isoamyl alcohol (25:24:1, *v*/*v*/*v*) and centrifuged for 45 min at 10,000 rpm at room temperature to separate the organic and the aqueous phases. To the upper phase, 0.2 volume of 1 M acetic acid and 0.7 volume of chilled alcohol were added to precipitate the RNA. This was incubated at −20 °C overnight. The RNA was pelleted by centrifugation at 10,000 rpm for 10 min at 4 °C. The pellet was then washed twice with 3 M sodium acetate (pH 5.2) to dissolve the low molecular weight RNA and contaminating polysaccharides. Subsequently, it was washed with 70% ethanol and dissolved in DEPC-treated sterile-distilled water. The RNA was quantified spectrophotometrically, and its quality was checked on a mini-agarose gel.

All solutions (except phenol, chloroform, acetic acid, guanidine buffer and ethanol) and plastic ware and glassware were treated with 0.1% and 0.01% DEPC (diethylpyrocarbonate), respectively, and autoclaved.

### 4.8. Electrophoresis and Northern Transfer

For 350 mL of 1.2% gel solution, 4.2 g of agarose was added to 339.5 mL of 1× MOPS (20 mM MOPS, 5 mM sodium acetate, 1 mM EDTA, pH 7.0) and treated with 0.1% DEPC for 1 h at room temperature. Agarose was melted and cooled by keeping it in a water bath set at 60 °C. To this 10.5 mL of 37% formaldehyde was added. The gel was allowed to solidify and submerged in 1× gel running buffer (MOPS).

To the RNA samples (100 µg in sterile-distilled water), 3 volumes of denaturation mix (formamide 500 µL, formaldehyde (37%) 62 µL, 10× MOPS, 100 µL) was added and mixed well and incubated at 65 °C for 10 min. To each sample, 0.2 volume of gel loading buffer was added and immediately kept on ice.

The samples were electrophoresed in agarose-formaldehyde gel at 120 V (constant voltage). After a 4 h run, the gel was washed in sterile-distilled water to remove the formaldehyde.

For northern transfer, the gel was blotted onto nitrocellulose paper, by capillary blotting in 20× SSC for 24 h. Subsequently, the gel was lifted and rinsed in 3× SSC and air-dried for 1 h and finally baked at 80 °C for 2 h.

The nitrocellulose filters containing the bound RNA were pre-hybridized in closed, shallow plastic trays containing 200 µL/cm^2^ prehybridisation solution (42% formamide, 5× SSC, 5× Denhardt’s solution (5 mg/mL of polyvinylpyrrolidone, bovine serum albumin and ficoll 400), 50 mM sodium phosphate buffer, pH 6.5, 250 µg/mL sonicated and denatured herring sperm DNA) for 16 h at 37 °C for nuclear genes or 42 °C for chloroplast encoded genes, in an incubator shaker at 60 rpm. Various gene-specific DNA probes were prepared from recombinant plasmids containing DNA fragments, either using electroeluted fragments or low melting point agarose containing the fragment. These were then labelled with the help of multiprime DNA labelling or megaprime DNA labelling system (Amersham International, Amersham, UK) using alpha ^32^P dATP, according to the manufacturer’s specifications. Spinach chloroplast DNA gene probes were used as described by Kapoor et al. [48], and nuclear gene probes for *RBCS* and *CAB* from Coruzzi et al. [49]. 25S rRNA gene probe was used as a control [50]. Double-stranded radiolabelled probes were denatured by boiling in a water bath for 3 min, followed by immediate cooling on ice for the electroeluted fragments, and the probes were added directly to the hybridisation buffer (23% formamide, 5× SSC, 1× Denhardt’s solution, 50 mM sodium phosphate, pH 6.5, 50 µg/mL sonicated and denatured herring sperm DNA). For the heterologous probes, 10% dextran sulphate was also added to the hybridisation mixture, the filters were then transferred to this after pre-hybridisation. Hybridisation was continued for 24–48 h in an incubator shaker at 37 °C or 42 °C, as per the requirement. The filters were first washed in solution 1 (42% formamide, 5× SSC, 0.1% SDS) at room temperature for 15 min. The second wash was given with 5× SSC and 0.1% SDS, at room temperature for 15 min, twice, and subsequently with 2× SSC and 0.1% SDS. The filters hybridized with chloroplast genes were washed with 2× SSC and 0.1% SDS at room temperature for 15 min, followed by 1× SSC and 0.1% SDS. When most of the non-specific radioactivity was removed, the filters were wrapped in cling film and exposed to the X-ray film (Konica, Tokyo, Japan) in a cassette containing an intensifying screen (Rege Chemicals, Delhi, India). The cassette was kept at −20 °C for exposure and the films developed after 24–72 h.

## Figures and Tables

**Table 1:** Biometry of morphological and anatomical parameters of the duckweed *Lemna gibba*, wild type (WT) and mutant (mt). * significance (*p* < 0.05). Data were given as Mean ± SD.

Parameter	Number of Samples	Wild Type	Mutant
Frond length (mm)	20	3.98 ± 0.11	6.31 ± 0.38 *
Frond width (mm)	20	3.12 ± 0.16	5.33 ± 0.37 *
Ratio frond length/width	20	1.28 ± 0.06	1.19 ± 0.06 *
Root length (cm)	20	1.20 ± 0.17	3.70 ± 0.59 *
Root diameter (µm)	43	161 ± 14	277 ± 30 *
Size of outer cortical cells of root (µm)	56	21.0 ± 4.9	40.8 ± 10.2 *

**Table 2:** Genome sizes of clones of *Lemna gibba*, clone WT 7796 and mt 9602 measured by flow cytometry. Genome sizes of three more clones of *L. gibba* collected from other regions were measured and presented for comparison. Data are given as Mean ± SD. For the detailed measurement data set see Supplementary Material, Table S1).

Clone ID	Origin	DNA Content (pg/C)	Genome Size (Mbp/1C)	Ploidy
WT (7796)	Italy	1.14 ± 0.02	557 ± 10	diploid
mt (9602)	Mutant of clone 7796	2.36 ± 0.05	1153 ± 26	tetraploid
Other *L. gibba* clones				
7641	Israel	1.16 ± 0.01	568 ± 6	diploid
7922	Argentina	1.09 ± 0.02	535 ± 8	diploid
8682	Saudi Arabia	1.15 ± 0.01	565 ± 3	diploid

**Table 3:** Growth rates of *Lemna gibba*, WT 7796 and mt 9602. Nutrient medium N and half-strength Hutner´s medium were used. *t* test (* significant different), *n* = 6.

Clone	RGR (d^−1^)	DT (d)	RY (g g^−1^ week^−1^)
N medium:			
WT	0.448 ± 0.004	1.56 ± 0.05	23.6 ± 2.9
mt	0.347 ± 0.040 *	2.03 ± 0.02	11.8 ± 3.2
Half-strength Hutner´s medium:			
WT	0.385 ± 0.021	1.80 ± 0.09	14.8 ± 0.8
mt	0.324 ± 0.009 *	2.14 ± 0.01	9.62 ± 0.29

RGR = relative growth rate; DT = doubling time; RY = relative weekly yield. For more details, refer to Section 4.

**Table 4:** Biochemical parameters of *Lemna gibba*, WT 7796 and mt 9602. Data are given per dry weight (DW). *n* = 4, means ± SD; * significant (*p* < 0.05).

Parameter	WT 7796	mt 9602
Dry weight (%)	5.30 ± 0.20	4.92 ± 0.29
Chlorophyll a (mg g^−1^ DW)	16.2 ± 1.82	8.78 ± 0.74 *
Chlorophyll b (mg g^−1^ DW)	6.05 ± 0.70	5.32 ± 0.91
Carotenoids (mg g^−1^ DW)	0.488 ± 0.060	0.203 ± 0.005 *
Protein content (% dry weight)	26.0 ± 0.1	22.8 ± 0.3 *

## Data Availability

All data are available within the manuscript or Supplementary Material.

## Supplementary Materials

The following supporting information can be downloaded at: https://www.mdpi.com/article/10.3390/plants12132525/s1, Table S1: List of individual genome size measurements (including the used cytometer and reference standard) performed for the *Lemna gibba* clones 7796, 9602, 7641, 7922 and 8682; Table S2: Nutrient media composition of N medium [36] and Hutner´s medium [37].
