# Supplementary material for: Characterisation of a Spontaneous Mutant of Lemna gibba G3 (Lemnaceae)
Source: Plants (Basel). 2023 Jul 2;12(13):2525. doi: 10.3390/plants12132525 (PMC10346275; doi:10.3390/plants12132525)
Supplement: Supplementary file 1 [file plants-12-02525-s001.zip › SupplementTableS2.pdf]

Supplement Table S2: Nutrient media N (Appenroth, K.J. et al., 1996, DOI10.1007/BF02879642) and Hutner´s medium (Hutner S.H., 1953: Comparative physiology of heterotrophic growth. In: LOOMIS W.E. (ed.), Growth and differentiation in plants. Iowa State, College Press, 417-446)

	N medium			Half-strength Hutner	
Salt	Formula	Molar concentration	Mass Concentration (mg L^{-1})	Molar concentration	Mass Concentration (mg L^{-1})
Ammonium nitrate	NH_4NO_3	-	-	1 mM	80
Boric acid	H_3BO_3	5 µM	0.309	0.121 mM	7.5
Calcium nitrate	$Ca(NO_3)_2$*$7H_2O$	1 mM	236	0.345 mM	100
Cobalt chloride	$CoCl_2$*$6H_2O$	-	-	2.1 µM	0.5
Copper sulphate	$CuSO_4$*$5H_2O$	-	-	8 µM	2
EDTA Iron sodium	FeNaEDTA	25 µM	9.2	-	-
EDTA Sodium	Na_2EDTA*$2H_2O$	-	-	0.672 mM	250
Iron sulphate	$FeSO_4$*$7H_2O$	-	-	50 µM	13.9
Magnesium sulphate	MgSO4*$7H_2O$	1 mM	246	1.02 mM	250

Manganese chloride	$MnCl_2$*4H_2O	13 µM	2.57	55 µM	10.9
Potassium dihydrogen phosphate	KH_2PO_4	150 µM	20.4	-	-
Di-Potassium hydrogen phosphate	K_2HPO_4	-	-	1.15 mM	200
Potassium nitrate	KNO_3	8 mM	809	-	-
Sodium molybdate	Na_2MoO_4*2H_2O	0.4 µM	0.097	57 µM	12.5
Zinc sulphate	$ZnSO_4$*7H_2O	-	-	113 µM	32.5
